# Difference in contagious yawning between *susceptible* men and women: why not?

**DOI:** 10.1098/rsos.160477

**Published:** 2016-09-07

**Authors:** Ivan Norscia, Elisa Demuru, Elisabetta Palagi

**Affiliations:** 1 Museo di Storia Naturale, Università di Pisa, Via Roma 79, Calci, Pisa 56011, Italy; 2 Unità di Primatologia Cognitiva, ISTC-CNR, via Aldrovandi 16b, 00197 Roma, Italy

**Keywords:** yawn contagion, gender bias, *Homo sapiens*

## 1. Comparing the incomparable

In their commentary, Gallup & Massen [1] criticize the fact that we did not consider ‘more than a dozen’ previous publications which did not report gender differences in human contagious yawning. We thank the authors for pointing out this issue and for giving us the possibility to provide a brief explanation on some aspects that are not as obvious as we thought.

Our investigation was ethological and our framework was centred on behavioural studies also on non-human primates and other mammals. We therefore selected the articles that were relevant to our comparative and evolutionary approach. Gallup & Massen [1] state that the gender difference in yawn contagion detected in our study is a false positive and that the null effect is real. Unfortunately, the sample that they used to make this assumption (17 negative cases and one positive case) is incorrect and, consequently, so is their conclusion. The possibility to find a phenomenon relies on whether the sample and the methodology used are suitable to detect it. To retain the metaphor used by Gallup & Massen [1], you can flip a coin as many times as you want and never find what you expect if what you expect is to get a six. You should change the approach and roll a dice, instead.

The results presented in the article by Palagi *et al.* [2] were based on naturalistic observations (and not on videos as it is said in table 1 of the commentary [1]) and the database also included bonobos, in which the sex of the trigger and not the sex of the responder tended to influence yawn contagion rates [3]. Therefore, it could not be used to evaluate which variables affect yawn contagion rates in humans only. Four of the articles mentioned in their commentary must be excluded from the sample, because they were focused on sexually immature subjects and on the difference between autistic versus non-autistic children [4–7], whereas our study was focused on non-pathological adults. Children are not suitable to test for gender differences because the power of empathy and yawn contagion is strongly influenced by age [8]. One further article cannot be included because the gender of the potential responder was not considered at all [9], and another one has to be excluded because it investigated yawn contagion and psychopathy [10]. In seven articles, the experimental subjects were either aware of the purpose of the study and/or a control condition was missing (the rate of spontaneous yawning) [11–17]. Adopting a blind procedure—with the experimental subjects not knowing the purpose of the study—is crucial when dealing with yawn contagion because simply thinking about yawning can elicit yawns [18]. Knowing the baseline level of spontaneous yawns is also pivotal to properly measure the real differences between the rate of spontaneous and infected yawns.

Four of the articles mentioned in Gallup & Massen's commentary [1] used static images as stimulus to elicit a motor pattern [12,14–16], and six were based on self-reports and not on objective observations (as the authors specify in table 1 of their commentary) [12–17]. The commentary's authors state that there is no *a priori* reason to believe that different methods and measurements would alter the expression of yawns in men versus women consistently in one direction. The literature, however, does not support this statement. Static images of facial expressions lack the dynamic complexity of naturalistic social–emotional interactions and, therefore, have limited external validity [19,20]. There is evidence that static and dynamic images have a different effect on men and women, with the latter showing an increase of the perception of the emotional intensity when exposed to both happy and angry dynamic facial stimuli [21]. As for the validity of self-reporting methods, a significant gender bias has been demonstrated in a wide variety of studies focusing on many different contexts [22–24]. Petrides & Furnham [25], for example, demonstrated gender differences in measured and self-estimated emotional intelligence with men showing higher correlations between measured and self-estimated scores, whereas women underestimated their emotional reactions and skills. Hence, there are solid reasons to believe that different methods and measurements can alter the expression of yawns in men versus women because the existence of methodology-related gender biases has already been highlighted in previous studies focusing on the expression of emotional states. If we exclude the articles that cannot be used for comparisons for the above-mentioned reasons (self-reported scores, static images, no proper control and non-blind procedures), only two articles of the initial pseudo-sample remain. These two studies considered humans in their natural conditions: one [26] was carried out on all individuals to find out what factors influenced the presence and frequency of yawn contagion and the other [27] considered only the susceptible population to detect if other factors could affect the rate of yawn contagion when yawn contagion occurs. Based on the real available sample, and the related probability, it cannot be stated that our result is a false positive.

## 2. Comparative versus comparable studies

As regards non-human animals, the commentary's authors criticize the fact that we did not cite all the literature taking into account possible sex effects in yawn contagion. This is not correct. We cited the literature that was relevant to support and understand our results. Very briefly, we excluded articles dealing with (i) birds [28] because the framework of our study connects yawn contagion with the mirror neuron system and the mammalian brain, (ii) stressed dogs showing no yawn contagion [29], (iii) animals exposed to videos of humans or avatars [30,31] and (iv) sexually immature subjects [32].

The work by Massen *et al*. [33] deserves a specific comment as it supports our general idea that yawn contagion is also influenced by the role that individuals play in their society. Chimpanzees form male-bonded societies. Hence, it is not surprising that yawn contagion may be higher in response to males, because the relationship with males can be the most meaningful to the group members. As we summarize in Norscia & Palagi [34], there is growing evidence that the social status affects the degree of emotional involvement of individuals and their interest in what others may feel [35,36]. However, we believe that this study should be replicated without using slow motion videos because, as said above, mimicry responses are influenced by the quality of motor patterns. Moreover, the authors failed to demonstrate that the yawning response of chimpanzees was elicited by the video stimulus and not by other group mates yawning nearby. This bias raises serious concerns on how to interpret the final results.

The other works mentioned by Gallup & Massen [1] were considered when and if appropriate. As we specify in our article, Campbell & de Waal [37] found that only the social bond influenced yawn contagion rates in chimpanzees, which is similar to what we found in humans in our previous article [26]. The same applies to the study on dogs by Romero *et al*. [38], which is cited in our article. The importance of social bond in influencing yawn contagion can be so strong as to dampen the effect of any other factor if we consider the whole population (both susceptible and non-susceptible subjects).

The commentary's authors also state that ‘the findings supporting a female bias in non-humans do not actually describe a female bias that is comparable to what Norscia and co-authors [27] report for humans'. However, we did not state that the other works of non-human animals described the exact same bias that we found. We stated that several other works had found a female skew (not the *same* skew) in yawn contagion and then we interpreted the different skews in the light of the role that females have in their groups ‘according to species-specific social dynamics’ ([27], p. 6). Finding exactly the same bias would go against our own framework, which links possible biases in yawn contagion to social dynamics. If the social dynamics are different, so should be the biases.

## 3. When the sample is not simple

The commentary's authors confirm that at least within our restricted sample women are more likely to yawn contagiously. And we still claim this. Within the susceptible subjects included in our study, women contagiously yawned more than men. In some of the studies cited in the commentary, some concerns could be raised about the possibility to generalize the results when the analyses are restricted to a certain cohort of individuals (e.g. undergraduate students), uprooted from their context (e.g. laboratory condition) and exposed to unreal stimuli (static images or slow motion videos; e.g. [11,16]).

In our case, Gallup & Massen [1] question how we selected the sample for the analysis, only leaving 34.5% of the original dataset. We indicated the size of the original dataset to precisely show that contrary to laboratory-controlled conditions, in natural settings it is necessary to gather an enormous quantity of behavioural bouts to obtain a sufficient amount of data suitable for analyses. This is a common situation in observational studies, not only in humans, but also in non-human animals.

It is true that in our 2016 study, we ‘did not assess whether there was a difference in contagious yawning frequency in the total sample of men and women’. Indeed, as Gallup & Massen [1] also note, we had already demonstrated in 2011 that in the susceptible and non-susceptible population there is no difference between men and women in the yawn contagion frequency [26]. As a further step, we wanted to verify whether within the population in which the phenomenon of yawn contagion is present, different factors other than social bonding would affect the rates of the yawning response. We confirmed that the social bond is a key factor but also that gender can make the difference in how much a subject responds to another one within the ‘yawning dyad’. Gallup & Massen [1] also criticize our choice of considering only the subjects exposed to at least three stimuli (yawns) but we strenuously defend this approach. In a natural setting, with confounding auditory and visual stimuli, we must be reasonably sure that the study stimulus is detected. This approach mirrors laboratory experimental procedures in which the yawning pattern is repeated several times in a single video to make sure that the stimulus is perceived by the observer.

In sum, the sample of articles on human contagious yawning that Gallup & Massen used to conclude that our result is a false positive is incorrect, because the cited articles cannot be reliably compared with our study. The comparative studies considering non-human animals were used to discuss why it is reasonable to interpret the different types of biases in contagious yawning in the light of the role that the individuals play in their social groups. Finally, not to replicate previously published results, we focused on the subjects that showed yawn contagion and we made sure that the eliciting stimulus was perceived. In our study, we found that women produce slightly more yawns than men (moderate effect size) and that gender plays a statistically significant role in susceptible people, with women showing a higher level of yawn contagion than men. We are confident that future studies will confirm our results.
